# Neuroprotective effects of osmotin in Parkinson’s disease-associated pathology via the AdipoR1/MAPK/AMPK/mTOR signaling pathways

**DOI:** 10.1186/s12929-023-00961-z

**Published:** 2023-08-11

**Authors:** Jun Sung Park, Kyonghwan Choe, Hyeon Jin Lee, Tae Ju Park, Myeong Ok Kim

**Affiliations:** 1 Division of Life Sciences and Applied Life Science (BK 21 Four), College of Natural Science, Gyeongsang National University, Jinju, 52828 Republic of Korea; 2 Department of Psychiatry and Neuropsychology, School for Mental Health and Neuroscience (MHeNs), Maastricht University, 6229ER Maastricht, the Netherlands; 3 Haemato-Oncology/Systems Medicine Group, Paul O’Gorman Leukaemia Research Centre, Institute of Cancer Sciences, College of Medical, Veterinary and Life Sciences (MVLS), University of Glasgow, Glasgow, G12 0ZD UK; 4 Alz-Dementia Korea Co., Jinju, 52828 Republic of Korea

**Keywords:** Parkinson’s disease, Osmotin, α-Synuclein, Dopaminergic neuron, Neuroinflammation

## Abstract

**Background:**

Parkinson’s disease (PD) is the second most frequent age-related neurodegenerative disorder and is characterized by the loss of dopaminergic neurons. Both environmental and genetic aspects are involved in the pathogenesis of PD. Osmotin is a structural and functional homolog of adiponectin, which regulates the phosphorylation of 5′ adenosine monophosphate-activated protein kinase (AMPK) via adiponectin receptor 1 (AdipoR1), thus attenuating PD-associated pathology. Therefore, the current study investigated the neuroprotective effects of osmotin using in vitro and in vivo models of PD.

**Methods:**

The study used 1-methyl-4-phenyl-1,2,3,6-tetrahydropyridine (MPTP)-induced and neuron-specific enolase promoter human alpha-synuclein (NSE-hαSyn) transgenic mouse models and 1-methyl-4-phenylpyridinium (MPP^+^)- or alpha-synuclein A53T-treated cell models. MPTP was injected at a dose of 30 mg/kg/day for five days, and osmotin was injected twice a week at a dose of 15 mg/kg for five weeks. We performed behavioral tests and analyzed the biochemical and molecular changes in the substantia nigra pars compacta (SNpc) and the striatum.

**Results:**

Based on our study, osmotin mitigated MPTP- and α-synuclein-induced motor dysfunction by upregulating the nuclear receptor-related 1 protein (Nurr1) transcription factor and its downstream markers tyrosine hydroxylase (TH), dopamine transporter (DAT), and vesicular monoamine transporter 2 (VMAT2). From a pathological perspective, osmotin ameliorated neuronal cell death and neuroinflammation by regulating the mitogen-activated protein kinase (MAPK) signaling pathway. Additionally, osmotin alleviated the accumulation of α-synuclein by promoting the AMPK/mammalian target of rapamycin (mTOR) autophagy signaling pathway. Finally, in nonmotor symptoms of PD, such as cognitive deficits, osmotin restored synaptic deficits, thereby improving cognitive impairment in MPTP- and α-synuclein-induced mice.

**Conclusions:**

Therefore, our findings indicated that osmotin significantly rescued MPTP/α-synuclein-mediated PD neuropathology. Altogether, these results suggest that osmotin has potential neuroprotective effects in PD neuropathology and may provide opportunities to develop novel therapeutic interventions for the treatment of PD.

**Supplementary Information:**

The online version contains supplementary material available at 10.1186/s12929-023-00961-z.

## Background

Parkinson’s disease (PD) is the second most common neurodegenerative disease and is clinically characterized by the loss of dopaminergic neurons and motor dysfunctions such as tremor, bradykinesia, muscle rigidity, and postural instability [14, 31]. Additionally, PD has nonmotor symptoms, including cognitive impairment [11]. At the molecular level, Lewy body aggregation, α-synuclein accumulation, disturbances in the ubiquitin‒proteasome system, neuroinflammation, and dysregulation of mitophagy occur [13]. The most susceptible neurons in PD are dopaminergic neurons in the substantia nigra pars compacta (SNpc) [42]. Chemical compounds, such as 1-methyl-4-phenyl-1,2,3,6-tetrahydropyridine (MPTP) and its active toxic molecule, 1-methyl-4-phenylpyridinium (MPP^+^), are a widely used to construct PD animal models since they can cross the blood‒brain barrier (BBB), disturb the mitochondrial transport chain and induce oxidative stress and the loss of dopaminergic neurons [4, 6]. Additionally, another PD model uses transgenic mice overexpressing human wild-type α-synuclein, and these mice exhibit several aforementioned characteristics of PD [30]. Recent findings have indicated that α-synuclein oligomers are the most toxic form of α-synuclein, and the secretion of these oligomers is important for the progression of PD [28]. Alterations, including increased oxidative stress, lipid abnormalities, complex I deficiency, loss of membrane potential, increased mitochondrial fragmentation, and the release of cytochrome c, have been reported for mutant α-synuclein (A53T and A30P) transgenic and wild-type α-synuclein-overexpressing cells [48].

Currently, there are no curative treatments for neurodegenerative diseases. One of the accepted hypotheses related to the pathophysiology of neurodegeneration is adiponectin deficiency [3]. Adiponectin is secreted by adipocytes and is a metabolic hormone that exerts antiatherogenic/glucose metabolism effects, enhances insulin sensitivity, and crosses the BBB to affect neurons via the adiponectin receptor (AdipoR) [47]. Previous studies have indicated that adiponectin and its homologs are neuroprotective against various neurodegenerative diseases and metabolic syndromes [3, 45, 50]. Moreover, several studies have suggested that adiponectin has a prominent role in brain metabolism and has neuroprotective effects against PD in cellular models [23, 43]. Osmotin (Os) is a 26-kDa multifunctional *Nicotiana tabacum*-derived protein that acts as a homolog of mammalian adiponectin [39]. Previous studies have suggested that osmotin acts as a ligand of AdipoR, inducing the phosphorylation of 5′ adenosine monophosphate-activated protein kinase (AMPK), an important energy sensor, and its downstream markers in several models to alleviate neuroinflammation, apoptosis, and neurodegeneration, which are associated with neurological and metabolic disorders [1, 3, 22, 44, 45, 53]. Therefore, in this study, we hypothesize that osmotin may reduce PD-associated neurodegeneration (dopaminergic neuronal cell death and neuroinflammation) and its clinical manifestations ([non]motor symptoms) by regulating multiple pathological features of PD.

## Methods

### Cell culture

Human neuroblastoma SH-SY5Y cells were purchased from Korea Cell Line Bank (KCLB, South Korea) and cultured in Dulbecco’s modified Eagle’s medium (DMEM; Gibco, NY, USA) containing a 1% antibiotic–antimycotic solution and 10% fetal bovine serum at 37 °C with 5% CO_2_. The mHippoE-14 embryonic mouse hippocampal cell line (Cedarlane, Canada) was derived at embryonic Day 14 and cultured in DMEM without sodium pyruvate. SH-SY5Y or BV-2 cells were treated with MPP^+^, SP600125, SB203580, and PD98059 (Sigma‒Aldrich, MO, USA).

### Plasmid transfection

The EGFP-alpha-synuclein-A53T plasmid was a gift from David Rubinsztein (Addgene #40823) [17]. Adiponectin receptor (AdipoR1) was knocked out by a commercial AdipoR1 CRISPR/Cas9-KO plasmid (Santa Cruz, CA, USA). The insertion of the puromycin gene as a selection marker was performed using an AdipoR1 HDR plasmid (Santa Cruz, CA, USA). Cells were transfected using the abovementioned plasmids and Lipofectamine 3000 (Invitrogen, CA, USA) according to the instructions provided by the manufacturer [53]. The cells were cultured, and the medium was removed 24 h before selection. A pure and stable pool of knockout cell lines was obtained by using 2.5 µg/ml puromycin, which was added to the growth medium as a selection marker.

### Animals and treatment

This study followed the Animal Research: Reporting of In Vivo Experiments (ARRIVE) guidelines. For MPTP treatment, male wild-type C57BL/6J mice (approximately 25–28 g, 7 weeks old) were purchased from Jackson Laboratory (Bar Harbor, ME, USA). C57BL/6-Tg (neuron-specific enolase promoter human alpha-synuclein [NSE-hαSyn]) Korl mice were obtained from the National Institute of Food and Drug Safety Evaluation (NIFDS, Cheongju, Korea). For validation purposes, the animals were genotyped according to the NIFDS PCR protocols using samples obtained from the tails. Animals were ordered in the same batch and litter and were handled as previously described [21]. Briefly, the mice were acclimated for one week under a 12-h dark/light cycle at 21 ± 2 °C with 60 ± 10% humidity and food and water ad libitum.

MPTP (Sigma‒Aldrich, MO, USA) was prepared in sterile distilled water and intraperitoneal (i.p.) injection at a dose of 30 mg/kg for five consecutive days, according to previously established guidelines [20]. C57BL/6J mice were randomly allocated into the following three groups: the control (CTL; vehicle-treated), MPTP, and MPTP + Os groups (*n* = 12 mice/group). C57BL/6-Tg (NSE-hαSyn) Korl mice were allocated into the following three groups: the wild-type (WT), NSE-hαSyn (α-syn), and α-syn + Os groups (*n* = 12 mice/group). The purification of osmotin has been previously described, and purified osmotin was injected at a dose of 15 mg/kg in saline [44]. MPTP mice were treated with intraperitoneal injections of osmotin two times a week for 5 weeks from 9 to 14 weeks of age. NSE-hαSyn mice develop deficits in motor performance at 13 months of age [41]. NSE-hαSyn mice were treated with i.p. injections of osmotin two times a week for 5 weeks from 9 to 14 weeks of age. After the behavioral tests, the mice were euthanized for the subsequent experiments.

### Open field test

For the open field test, the mice were monitored in an open field box (40 × 40 cm with a height of 40 cm), which was divided into 16 equal-sized squares. The tests were conducted in a sound-reduced room under low light to prevent distractions and unintentional freezing behaviors. The trials were individually initiated when a mouse was placed into the center of the apparatus. The parameters used for analysis were the total distance traveled and the time spent in the central area. All of the experimental data were recorded with a SMART video tracking system (Panlab, MA, USA).

### Pole test

The pole was a rough wooden stick that was 40 cm in length and 10 mm in diameter. Before the tests, the mice were acclimated to the behavioral room and received 3 training trials per day for two consecutive days. The mice were placed on the top of the vertical wooden stick with the head in the face-up position. The total time (T-LA) taken to arrive at the bottom of the pole and place the forefeet on the floor was noted. The results are described as the T-LA latency (% of control), and the obtained results were compared among the experimental groups.

### Wire hang test

In the wire hang test, the mice hung onto a thin wire with their forelimbs, and the latency to fall was recorded. The mice were acclimated in the behavioral room before the experiment and were mounted 20 cm above the ground surface on a thin stretched wire. The test was repeated eight times for each group with the mice resting between several trials. The results are described as the latency to fall in seconds (sec).

### Morris water maze test

A Morris water maze (MWM) apparatus was used, which consisted of a circular water tank (100 cm in diameter and 40 cm in height) containing opaque water (23 ± 1 °C) at a depth of 15.5 cm. A transparent escape platform was hidden below the water surface and placed at the midpoint of one quadrant. Each mouse received training for five consecutive days. The latency to escape from the water maze, which was determined as successfully finding the hidden escape platform, was calculated for each trial. A probe test was performed to evaluate memory consolidation after compelling the mice to swim freely for 60 s without access to the escape platform. All data were automatically recorded using SMART video tracking software (Panlab, MA, USA).

### Antibodies

The following primary antibodies were used: TH and NeuN (Merck-Millipore, MA, USA); Bcl-2, Bax, Cytochrome c, Caspase-3, PARP-1, phospho-JNK, JNK, Nurr1, DAT, GFAP, Iba-1, PSD-95, SNAP-25, Synaptophysin (SYP), phospho-mTOR (296. Ser2481) and β-actin (Santa Cruz, CA, USA); AMPKα, phospho-AMPKα, phospho-p44/42 MAPK (ERK1/2, Thr202/Tyr204), p44/42 MAPK (ERK1/2), phospho-p38 MAP kinase (Thr180/Tyr182), p38 MAPK, α-synuclein, phospho-CREB (Ser133), CREB, and mTOR (Cell Signaling, MA, USA); VMAT2 and phospho-α-synuclein (Ser129) (Abcam, Cambridge, UK); and phospho-α-synuclein (Ser129, BioLegend, CA, USA). Detailed antibody information is provided in Additional file 1: Table S1.

### Immunofluorescence assays

Immunofluorescence analysis was performed as previously described with modifications [22]. Tissue sections were incubated with primary antibodies and treated with secondary antibodies conjugated with Alexa Fluor 488 or 594 (Invitrogen, CA, USA). For nuclear staining, the sections were stained with 4′,6′-diamidino-2-phenylindole (DAPI) and mounted with fluorescence mounting medium (Agilent, CA, USA). Images were taken with a confocal laser-scanning microscope (FluoView FV1000MPE, Olympus, Tokyo, Japan).

### Measurement of reactive oxygen species (ROS)

The ROS levels were evaluated in the brain tissue samples by measuring the oxidation of 2,7-dichlorodihydrofluorescein diacetate (DCFDA, Santa Cruz, TX, USA) to 2,7-dichlorofluorescein (DCF). The conversion was evaluated with a spectrofluorometer (Promega, WI, USA) using an excitation wavelength of 484 nm and an emission wavelength of 530 nm. For background fluorescence analysis (conversion of DCFH-DA in the absence of homogenate), parallel blanks were maintained. The results are presented as a histogram generated with GraphPad Prism 8.

### Reverse transcription-polymerase chain reaction (RT‒PCR)

Cells were prepared and analyzed using RT‒PCR in accordance with the manufacturer’s instructions. The primer sequences used for this analysis were as follows: SNCA: forward, 5′-TGT AGG CTC CAA AAC CAA GG-3′ and reverse, 5′-TGT CAG GAT CCA CAG GCA TA-3′. The RT‒PCR experiments were performed in triplicate.

### ApoTox-Glo Triplex assay

The ApoTox-Glo Triplex Assay (Promega, WI, USA) was used to estimate cell viability, cytotoxicity, and caspase-3/7 activation (apoptosis) and was performed as previously described [44]. The absorbance and fluorescence values were measured using a microplate spectrophotometer and the GloMAX Multi Detection System (Promega, WI, USA).

### Flow cytometry

SH-SY5Y cells (5 × 10^5^ cells) were harvested and then stained using an Annexin V-PE apoptosis detection kit (Abcam, Cambridge, UK) to assess the proportion of apoptotic cells. The stained cells were analyzed by a FACSVerse flow cytometer (BD, NJ, USA) and FlowJo V10 software (FlowJo, OR, USA). The experiments were conducted under the same experimental conditions with 20,000 cells per group.

### Golgi staining and morphological analysis of pyramidal cells

Golgi staining was performed as previously described with minor modifications [53]. Brain tissue was extracted from WT and NSE-hαSyn transgenic mice treated with either vehicle or osmotin. In a blinded manner, fifteen neurons per sample were selected and analyzed. Three hundred dendritic segments per group were designated randomly from the apical and basal regions of CA1 pyramidal neurons and examined to classify dendritic spine density. Dendritic complexity was evaluated using the FD Rapid GolgiStain kit (FD NeuroTechnologies, MD, USA) according to the manufacturer’s instructions. Sections (150–200 µm) were examined using a Leica DM6500B light microscope (Leica, Wetzlar, Germany) and an Axioskop-2-plus microscope (Zeiss, Oberkochen, Germany). Neuronal morphology was analyzed with ImageJ software using Sholl analysis. The spines were categorized according to their shape as filopodia-like spines, thin spines, stubby spines, or mushroom spines. The length of an individual spine was measured from the tip to the stalk of the dendrite using ImageJ software.

### Nissl staining/cresyl violet staining

Nissl staining was performed according to established protocols with modifications [21]. The sections were incubated with 0.1% cresyl violet solution (Sigma‒Aldrich, MO, USA) and washed with 70% and 100% ethanol for dehydration. Images were captured using an Olympus AX70 microscope (Olympus, Tokyo, Japan).

### Statistical analysis

The data were analyzed using GraphPad Prism 8, version 8.0.2. Statistical tests were determined based on the outcome of the normality test. Statistical data are presented as the mean ± standard deviation (SD) based on at least three independent experiments. For morphological analysis, three images from at least three independent experiments were considered. One-way ANOVA was performed with Bonferroni post hoc analysis, and *p* < 0.05 was considered statistically significant.

## Results

### Osmotin ameliorates MPTP/α-synuclein-induced behavioral and motor deficits

**Fig. 1:**
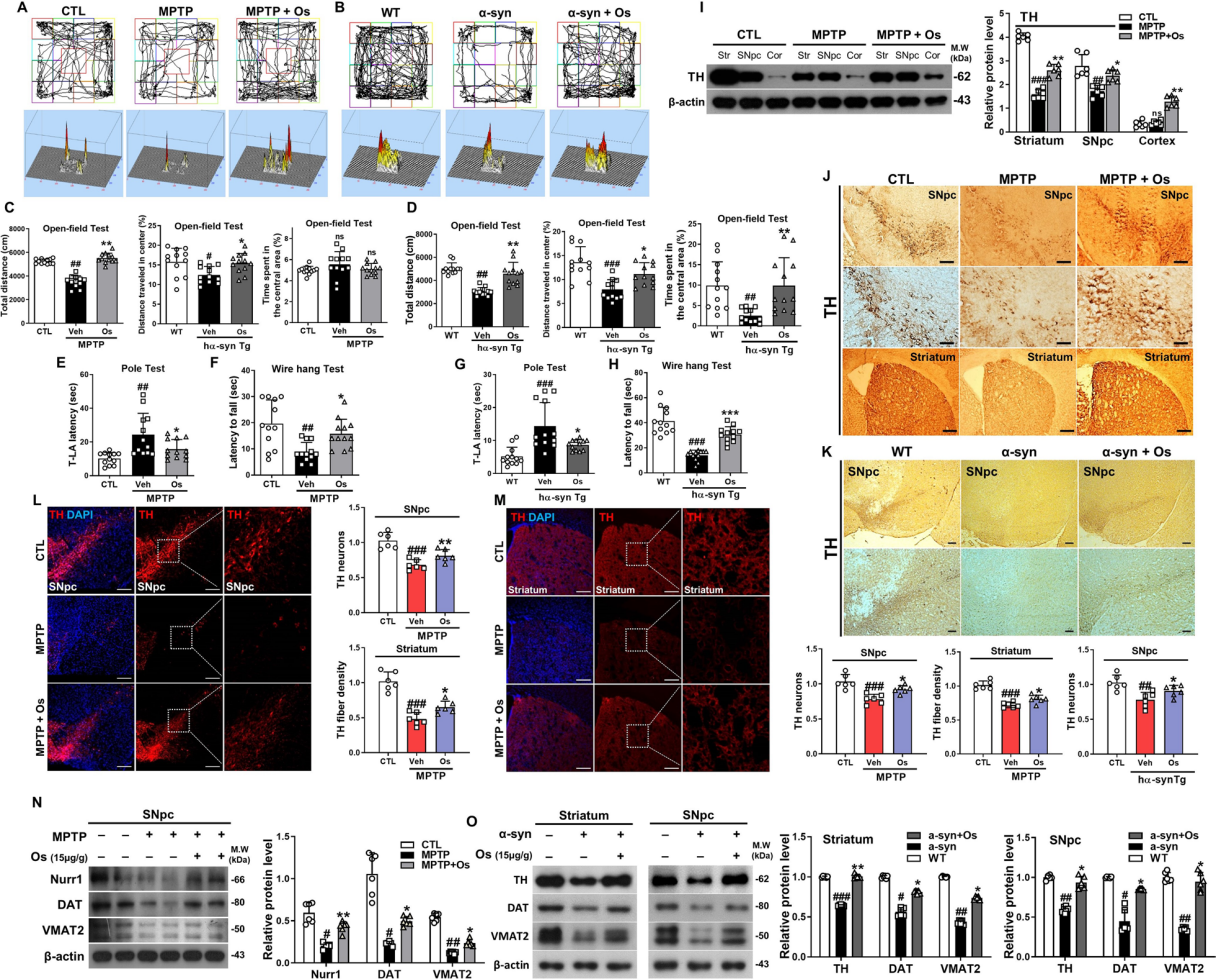
Osmotin ameliorates motor dysfunction and upregulates dopaminergic markers. **a**, **c** An open field test was performed on MPTP-induced mice (*n* = 12, biologically independent animals). **b**, **d** An open field test was performed on NSE-hαSyn Tg mice (*n* = 12, biologically independent animals). **a**, **b** Top panels represent an illustrative example of the open field test travel pathway and bottom panel represent an example of the global activity map in the open field test of the three groups. **e**, **f** Pole and wire hang tests were performed on MPTP-induced mice (*n* = 12, biologically independent animals). **g**, **h** Pole and wire hang tests were performed on NSE-hαSyn Tg mice (*n* = 10, biologically independent animals). **i** Western blot analysis of TH expression in MPTP-induced model mice (*n* = 6, biologically independent animals). **j**, **k** Representative images of coronal sections from MPTP/NSE-hαSyn Tg mice showing TH-positive neurons and striatal sections stained for TH immunoreactivity (*n* = 6, biologically independent animals). Scale bar represents 100 μm. **l**, **m** Immunofluorescence analysis of TH immunostaining in the SNpc and striatum of MPTP-induced mice (*n* = 6, biologically independent animals). Scale bar represents 100 μm. **n** Immunoblot results of Nurr1, DAT, and VMAT2 in the experimental groups and a graphical illustration (*n* = 6, biologically independent animals). **o** Immunoblot results of TH, DAT, and VMAT2 in the striatum and SNpc of NSE-hαSyn Tg mice and the respective bar graphs (*n* = 6, biologically independent animals). The data are presented as the mean ± SD and are representative of three independent experiments performed in triplicate. Significance was determined by using one-way ANOVA with Bonferroni correction; ^#^Comparison between control and MPTP/NSE-hαSyn Tg mice, *Comparison between MPTP/NSE-hαSyn Tg mice and osmotin-administered mice. ^#/^*p < 0.05, ^##/^**p < 0.01, and ^###/^***p < 0.001

We investigated the protective effects of osmotin against motor deficits in MPTP-induced and NSE-hαSyn mice. We observed significant motor impairment in the model mice, as determined by several behavioral tests. In the open-field test, compared to control mice, MPTP and NSE-hαSyn mice showed a significant reduction in the total distance covered. However, these effects were significantly reversed in the osmotin-treated group (Fig. 1a–d). Additionally, there was no difference between the MPTP treatment and control groups in terms of the time spent in the central area (Fig. 1c). Next, the effects of osmotin on bradykinesia and neuromuscular strength were assessed. In the pole test, compared to the control group, the MPTP/NSE-hαSyn groups showed increased total time (T-LA) to return to the floor, but osmotin treatment reduced the total time (Fig. 1e and g). In the wire-hang test, the MPTP/NSE-hαSyn groups had a reduced latency to fall, but osmotin treatment increased the latency to fall (Fig. 1f and h). Overall, these results suggest that osmotin ameliorates motor deficits in the MPTP/NSE-hαSyn groups.

### Osmotin protects against PD-associated pathologies in vitro and in vivo

Prior to evaluating the effects of osmotin against MPP^+^/A53T-induced toxicity in vitro, we conducted cell viability, cytotoxicity, and caspase-3/7 activity analyses at different doses and determined that 2.5 mM MPP^+^ for 24 h was the optimum dose for evaluating the effects of osmotin (Additional file 1: Figure S1a). Once the MPP^+^ dose was determined, we analyzed the impact of osmotin at doses of 5, 10, 15, and 20 µg/ml, and the results indicated that all four doses were optimal for adequate neuroprotective effects (Additional file 1: Figure S1b). In a similar manner, we also measured the effects of osmotin on the A53T-induced cell line and osmotin exerted neuroprotective effects (i.e., increased cell viability and decreased cytotoxicity and apoptosis) (Additional file 1: Figure S1c).

Next, the effect of osmotin against PD-associated pathologies such as α-synuclein accumulation and dopaminergic neuronal loss was investigated. Our findings suggested that the TH level was significantly reduced in the MPTP/NSE-hαSyn mice compared to control mice and was restored after osmotin administration (Fig. 1j and k). Moreover, osmotin significantly enhanced the number of TH-positive neurons in the SNpc and striatum of MPTP-induced mice (Fig. 1l and m). Similarly, our western blot results showed downregulated TH expression in MPTP/NSE-hαSyn mice, which was upregulated in osmotin-treated mice (Fig. 1i and o). Furthermore, the levels of other dopaminergic neuronal-related markers, such as Nurr1, VMAT2, DAT, and TH, in the SNpc and striatum of MPTP/NSE-hαSyn mice were significantly increased by osmotin treatment (Fig. 1i, n and o; Additional file 1: Figure S2a and b). Additionally, we validated the in vivo findings in MPP^+^-induced SH-SY5Y cells (Additional file 1: Figure S2c). Finally, to investigate the previously hypothesized mode of action of osmotin through AdipoR1, we established a CRISPR/Cas9-mediated AdipoR1-knockout (AKO) mouse hippocampal cell line. Immunofluorescence and immunoblot analysis showed that osmotin had no effect on AdipoR1 or AMPK, a downstream target of AdipoR1, to reduce α-synuclein when the AKO cell line was co-transfected with the A53T overexpression plasmid (Additional file 1: Figure S3a and b). Moreover, the protein expression of TH, DAT, and VMAT2 was also downregulated in the AKO cell line even after osmotin treatment (Additional file 1: Figure S3c). Collectively, our findings suggest that osmotin may protect dopaminergic neurons from PD pathology-induced neurotoxicity.

### Osmotin alleviates cell damage and reduces α-synuclein accumulation

**Fig. 2:**
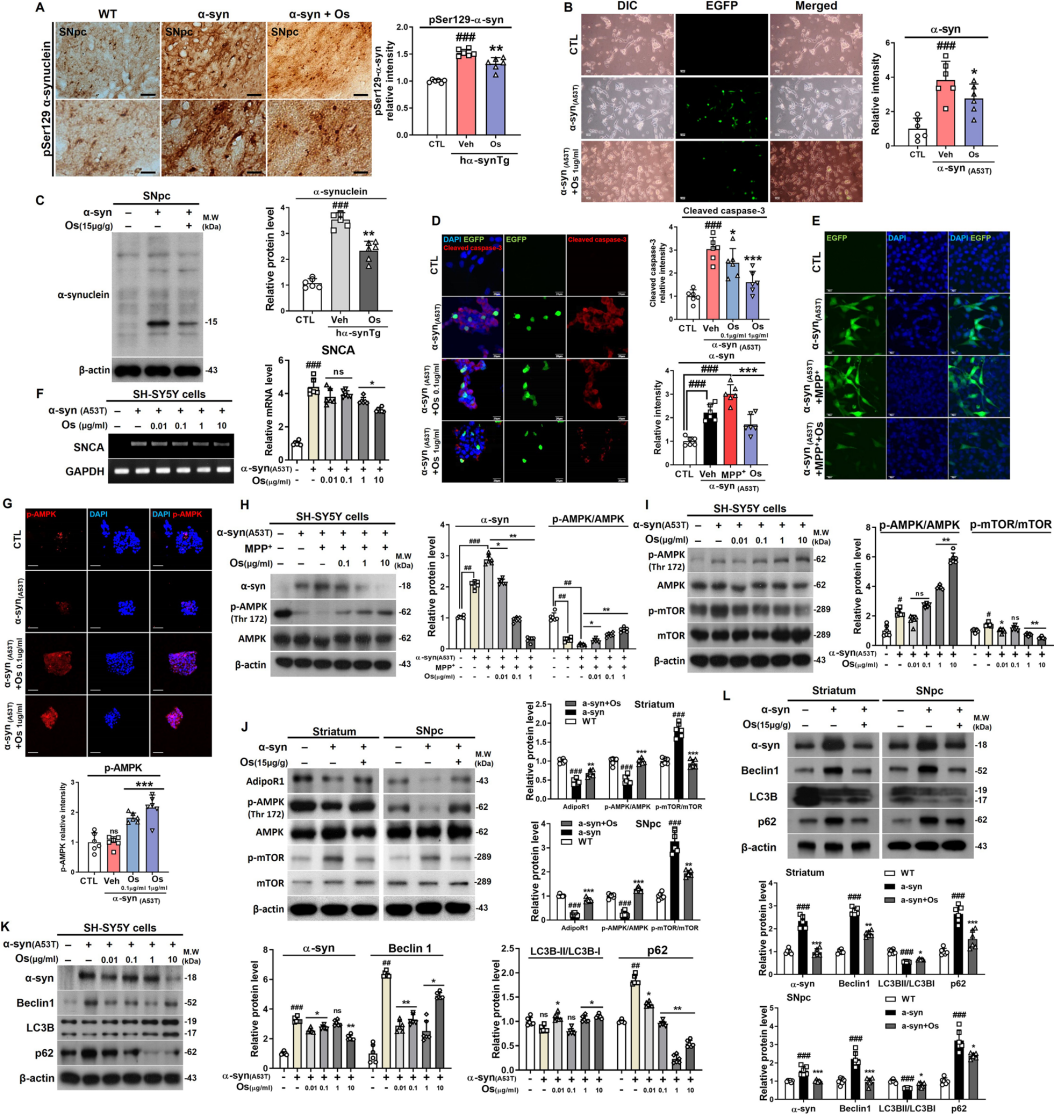
Osmotin protects against α-synuclein-induced pathology in vivo and in vitro. **a** Immunostaining for p-Ser129-α-synuclein in the SNpc of NSE-hαSyn Tg mice (*n* = 6, biologically independent animals). Scale bar represents 20 μm. **b** Representative immunostaining of EGFP in α-synuclein (A53T)-transfected SH-SY5Y cells. Scale bar represents 50 μm. **c** Immunoblot results of α-synuclein in the SNpc of NSE-hαSyn Tg mice (*n* = 6, biologically independent animals). **d** Immunofluorescence images of cleaved caspase-3 in α-synuclein (A53T)-transfected SH-SY5Y cells. Scale bar represents 20 μm. **e** Immunofluorescence images of EGFP in α-synuclein (A53T)-transfected/MPP^+^-induced SH-SY5Y cells. Scale bar represents 20 μm. **f** The degree of activation of SNCA was determined by RT‒PCR. **g** Immunofluorescence images of p-AMPK in α-synuclein (A53T)-transfected SH-SY5Y cells. Scale bar represents 20 μm. **j** Immunoblot results of AdipoR1, p-AMPK, AMPK, p-mTOR, mTOR in the striatum and SNpc of NSE-hαSyn Tg mice (*n* = 6, biologically independent animals). **h** Immunoblot results of α-synuclein, p-AMPK, and AMPK in α-synuclein (A53T)-transfected/MPP^+^-induced SH-SY5Y cells. **i**, **k**, **l** Immunoblot results of p-AMPK, AMPK, p-mTOR, mTOR, α-synuclein, Beclin1, LC3B, and p62 levels in striatum and SNpc of NSE-hαSyn mice and α-synuclein (A53T)-transfected SH-SY5Y cells. The data are presented as the mean ± SD and are representative of three independent experiments performed in triplicate. Significance was determined by using one-way ANOVA with Bonferroni correction; ^#^Comparison between control and MPTP/NSE-hαSyn Tg mice, *Comparison between MPTP/NSE-hαSyn Tg mice and osmotin-administered mice. ^#/^*p < 0.05, ^##/^**p < 0.01, and ^###/^***p < 0.001

Next, the effect of osmotin on the accumulation of α-synuclein aggregates was assessed. The Lewy body-like pathology induced by pSer129-α-synuclein in the SNpc of the NSE-hαSyn mice was significantly decreased with the administration of osmotin (Fig. 2a). Additionally, the expression of α-synuclein in the SNpc of NSE-hαSyn mice and A53T-transfected cells was significantly downregulated by osmotin (Fig. 2b, c and f). Furthermore, osmotin significantly downregulated the expression of cleaved caspase-3, an apoptosis-related protein, compared to its expression in NSE-hαSyn mice (Fig. 2d). Moreover, immunofluorescence staining showed that osmotin increased the phosphorylation of AMPK (p-AMPK) in A53T-transfected cells (Fig. 2g). Next, we analyzed the expression of α-synuclein and p-AMPK in MPP^+^-induced A53T-transfected cells, and the results showed that osmotin significantly reduced α-synuclein levels and activated AMPK phosphorylation (Fig. 2e, h and i). Furthermore, dysfunction of the autophagy-related pathways have shown in both brains of animal models and patients with PD [35]. Therefore, to investigate whether the osmotin-induced reduction of α-synuclein levels in A53T-transfected cells and NSE-hαSyn mice was mediated by autophagy, we analyzed the levels of autophagy-related markers in the experimental groups. We analyzed the phosphorylation of mammalian target of rapamycin (mTOR), an autophagy switching protein, in A53T-transfected cells, and the results showed that osmotin significantly inhibited p-mTOR (Fig. 2i). We analyzed the AdipoR1 and phosphorylation of mTOR, an autophagy switching protein, in striatum and SNpc of NSE-hαSyn mice, and the results showed that osmotin also significantly inhibited p-mTOR (Fig. 2j). Furthermore, we also measured the levels of Beclin1, LC3B, and p62, which are key factors in autophagy, and the results indicated that osmotin rescued autophagy dysfunction in striatum and SNpc of NSE-hαSyn which was validated in A53T-transfected cells (Fig. 2k, l).

### Osmotin regulates MPTP/α-synuclein-induced apoptosis

**Fig. 3:**
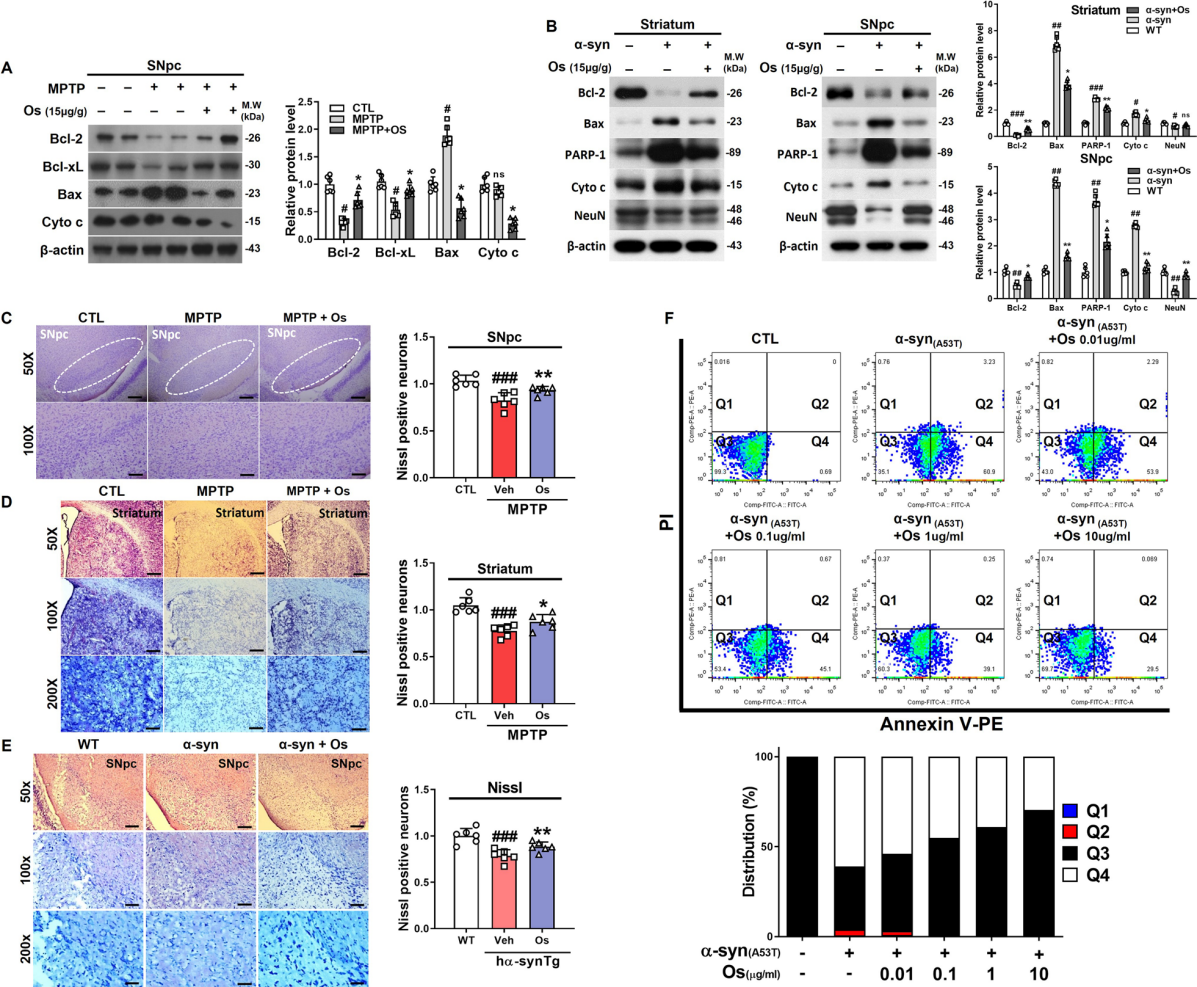
Osmotin regulates MPTP/α-synuclein-induced apoptosis in vivo and in vitro. **a** Immunoblot results of Bcl-2, Bcl-xL, Bax, and cytochrome c levels in the SNpc of MPTP-induced mice (*n* = 6, biologically independent animals). **b** Immunoblot results of Bcl-2, Bax, PARP-1, cytochrome c, and NeuN in the striatum and SNpc of NSE-hαSyn Tg mice (*n* = 6, biologically independent animals). **c, d** Representative photomicrograph of coronal mesencephalon and striatal sections from MPTP-induced mice containing Nissl-positive neurons (*n* = 6, biologically independent animals). Magnification: 200×, 100×, 50×. Scale bar represents 80 μm, 40 μm, 20 μm. **e** Representative photomicrograph of coronal mesencephalon sections from NSE-hαSyn Tg mice containing Nissl-positive neurons (*n* = 6, biologically independent animals). Magnification: 200X, 100X, 50X. Scale bar represents 80 μm, 40 μm, 20 μm. **f** Flow cytometric analysis of Annexin V-PE apoptosis detection assays in α-synuclein (A53T)-transfected SH-SY5Y cells. The data are presented as the mean ± SD and are representative of three independent experiments performed in triplicate. Significance was determined by using one-way ANOVA with Bonferroni correction; ^#^Comparison between control and MPTP/NSE-hαSyn Tg mice, *Comparison between MPTP/NSE-hαSyn Tg mice and osmotin-administered mice. ^#/^*p < 0.05, ^##/^**p < 0.01, and ^###/^***p < 0.001

To investigate whether osmotin inhibits MPTP/MPP^+^-induced neuronal cell death, pro-/anti-apoptotic markers were investigated. Western blot analyses showed that MPTP significantly increased Bax and decreased Bcl-2 and Bcl-xL levels in the SNpc. In contrast, osmotin reduced the level of Bax and cytochrome c release and increased Bcl-2 and Bcl-xL levels (Fig. 3a). Additionally, the immunofluorescence results suggested that osmotin markedly downregulated the expression of caspase-3 in the SNpc of MPTP-induced mice (Additional file 1: Figure S4a). These in vivo findings were validated in the in vitro model (Additional file 1: Figure S4b). We also evaluated the anti-apoptotic effects of osmotin against apoptotic cells and showed that osmotin significantly downregulated the expression of Bcl-2, Bax, cytochrome c, and PARP-1 in the striatum and SNpc of NSE-hαSyn mice, which was validated in an in vitro model (Fig. 3b; Additional file 1: Figure S4c). Moreover, Nissl staining and immunofluorescence analysis of NeuN immunostaining was used to analyze MPTP/α-synuclein-induced neurotoxic effects in the SNpc (Fig. 3c and e, Additional file 1: Fig. S5b, c) and striatum (Fig. 3d, Additional file 1: Fig. S5a), and showed reduction in the number of neurons, while osmotin treatment increased the number of neurons. Finally, we performed flow cytometry analysis with Annexin V-PE staining to measure the proportion of apoptotic A53T-transfected SH-SY5Y cells. The percentage of early apoptotic cells (Q4) increased from 0.69% in the control group to 60.9% in the A53T-transfected group. In contrast, osmotin suppressed the early apoptosis rates in a concentration-dependent manner, whereas osmotin slightly decreased the late apoptosis rate (Q2) compared with that of the A53T-transfected group (Fig. 3f). Based on these results, osmotin treatment showed neuroprotective effects against apoptosis in PD.

### Osmotin regulates stress responses and neuroinflammation

**Fig. 4:**
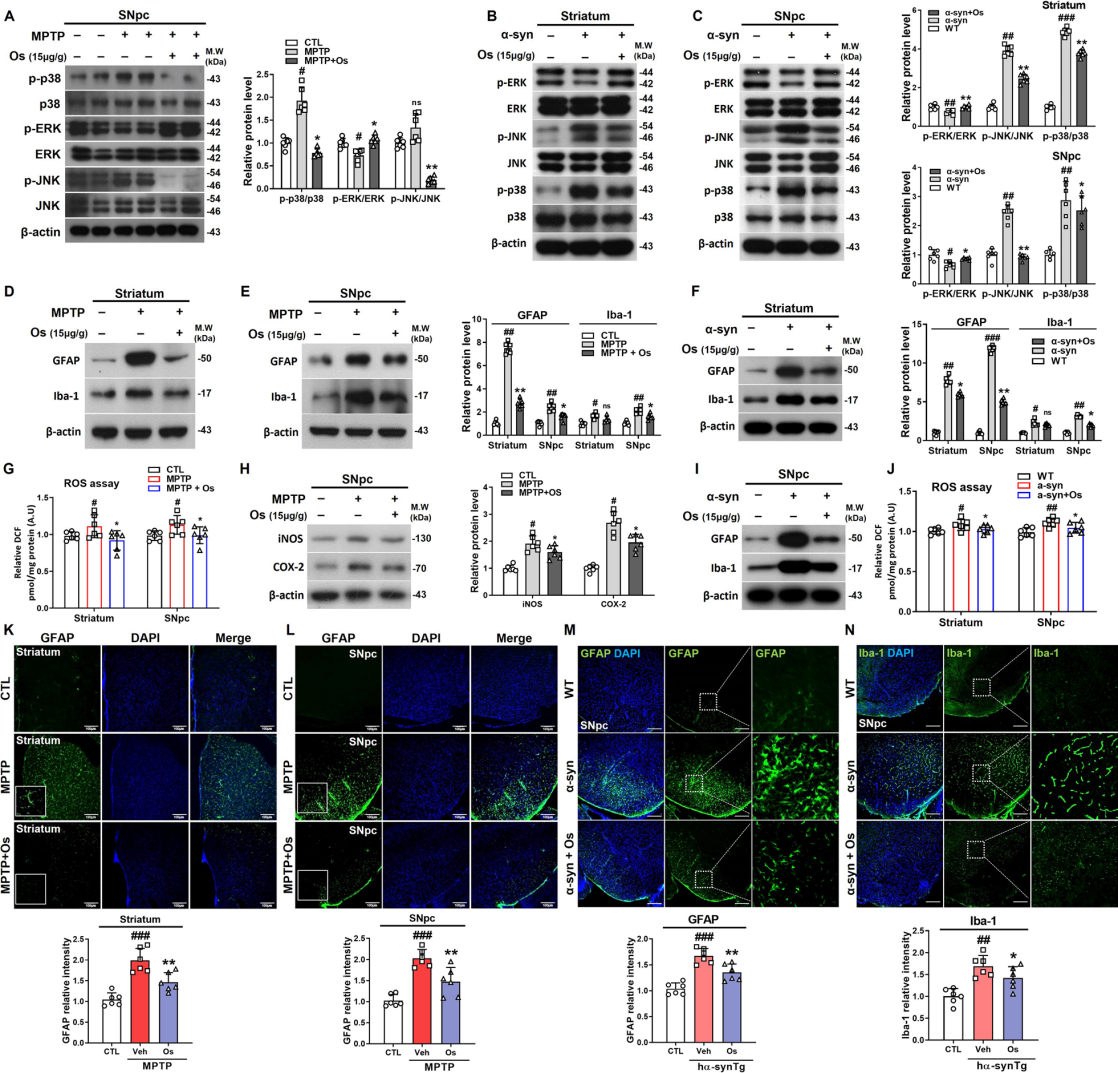
Osmotin regulates the phosphorylation of MAPK and neuroinflammation in MPTP/NSE-hαSyn Tg PD mice. **a** Immunoblot results of p-p38, p38, p-ERK, ERK, p-JNK, and JNK in the SNpc of MPTP-induced mice (*n* = 6, biologically independent animals). **b, c** Immunoblot results of p-ERK, ERK, p-JNK, JNK, p-p38, and p38 in the striatum and SNpc of NSE-hαSyn Tg mice (*n* = 6, biologically independent animals). **d, e** Immunoblot results of GFAP and Iba-1 levels in the striatum and SNpc of MPTP-induced mice (*n* = 6, biologically independent animals). **g** Representative graphs showing ROS levels in the striatum and SNpc of MPTP-induced mice. **h** Immunoblot results of iNOS and COX-2 expression in the SNpc of the MPTP-induced mice (*n* = 6, biologically independent animals). **f**, **i** Immunoblot results of GFAP and Iba-1 levels in the striatum and SNpc of NSE-hαSyn Tg mice (*n* = 6, biologically independent animals). **j** Representative graphs showing ROS levels in the striatum and SNpc of NSE-hαSyn Tg mice. **k**, **l** Representative immunofluorescent images of GFAP in the striatum expression and SNpc of MPTP-induced mice (*n* = 6, biologically independent animals). Scale bar represents 100 μm. **m**, **n** Representative immunofluorescent images of GFAP and Iba-1 levels in the SNpc of NSE-hαSyn Tg mice (*n* = 6 biologically independent animals). Scale bar represents 100 μm. The data are presented as the mean ± SD and are representative of three independent experiments performed in triplicate. Significance was determined by using one-way ANOVA with Bonferroni correction; ^#^Comparison between control and MPTP/NSE-hαSyn Tg mice, *Comparison between MPTP/NSE-hαSyn Tg mice and osmotin-administered mice. ^#/^*p < 0.05, ^##/^**p < 0.01, and ^###/^***p < 0.001

The mitogen-activated protein kinase (MAPK) family proteins p38, JNK, and ERK are involved in stress-activated responses and linked to cell survival and neuroprotection [18]. Since studies have shown dysfunction of the MAPK protein in PD [8], we analyzed the effects of osmotin on MAPK-associated pathways and investigated the expression of p-p38, p-JNK, and p-ERK in MPTP/α-synuclein-induced models in vivo and in vitro. According to our findings, there was upregulated expression of p-p38 and p-JNK and downregulated expression of p-ERK in MPTP/NSE-hαSyn mice, but osmotin treatment significantly reversed this effect (Fig. 4a–c). Furthermore, in vitro studies supported the in vivo results in a dose-dependent manner (Additional file 1: Figure S6a and b). To further elucidate the link between MAPK signaling and inflammation under osmotin treatment, we treated BV-2 cells with JNK (SP600125), p38 (SB203580), and ERK inhibitors (PD98059) under MPP^+^ treatment (Additional file 1: Figure S6c). We also evaluated the effects of osmotin against activated astrocytes (GFAP) and microglial cells (Iba-1) to determine its impact on the neuroinflammatory response. Our findings showed that the expression levels of both proteins were elevated in the striatum and SNpc of MPTP/NSE-hαSyn mice and were markedly reduced by osmotin treatment (Fig. 4d–f and i), and immunofluorescence results supported these findings (Fig. 4k–n; Additional file 1: Figure S7). These findings were confirmed by the upregulated expression of iNOS, a key proinflammatory enzyme, and COX-2, a key inflammatory response enzyme, in the SNpc of the MPTP-induced mice, and these levels were subsequently reduced by the administration of osmotin (Fig. 4h). Finally, to investigate the possible role of oxidative stress, we performed an ROS assay. The findings suggested that the ROS level was significantly reduced in the osmotin-treated mice compared to the MPTP/NSE-hαSyn mice (Fig. 4g and j).

### Osmotin regulates dendritic complexity and structure and increases spine density in pyramidal neurons

**Fig. 5:**
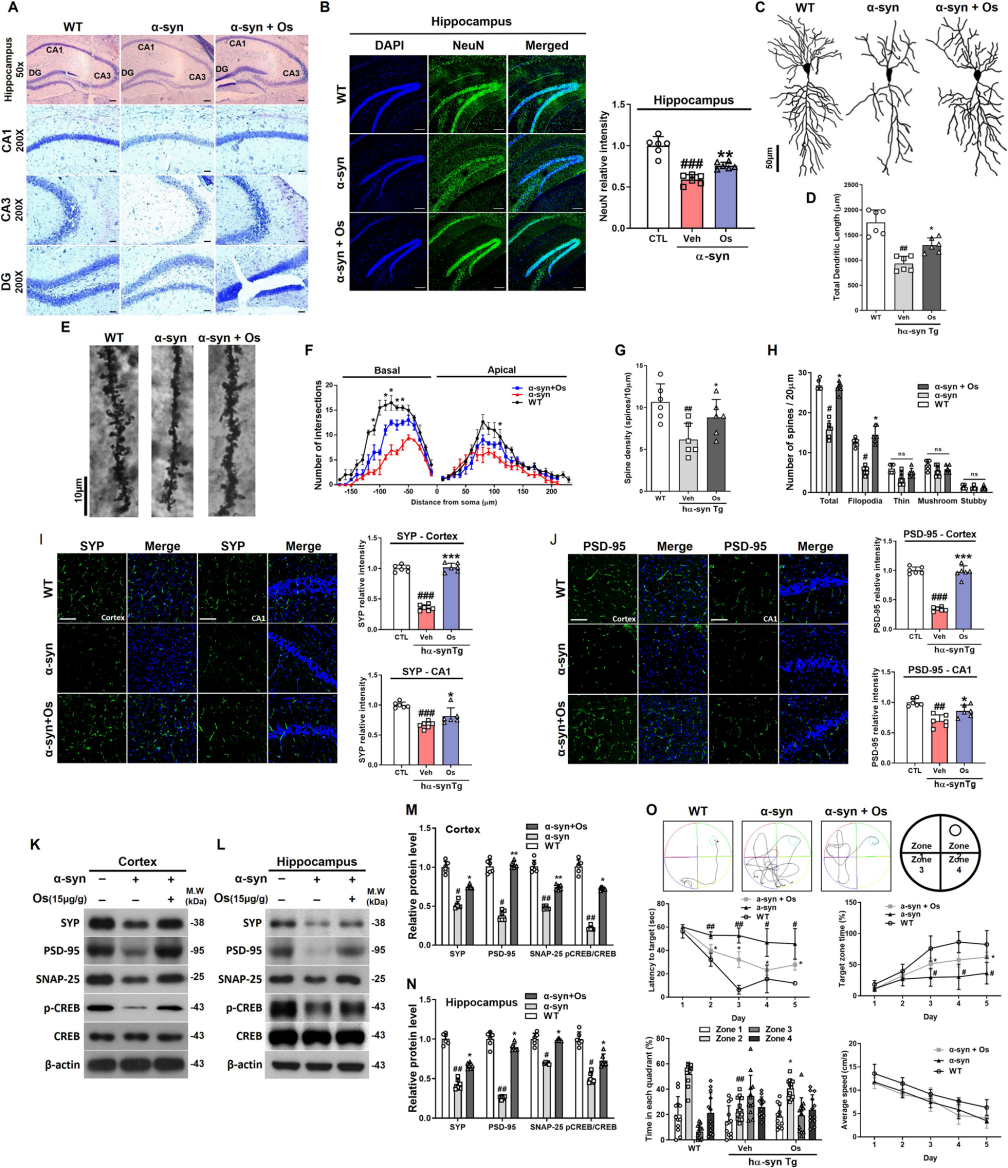
Osmotin restores the synaptic structures in pyramidal neurons and ameliorates cognitive deficits. **a** Representative photomicrograph of hippocampal (CA1, CA3, and DG) sections containing Nissl-positive neurons from NSE-hαSyn Tg mice (*n* = 6, biologically independent animals). Magnification: 50×. Scale bar represents 40 μm. Magnification: 200×. Scale bar represents 10 μm. **b** Representative immunofluorescent images of NeuN levels in the hippocampus regions of NSE-hαSyn Tg mice (*n* = 6, biologically independent animals). Scale bar represents 100 μm. **b** Representative immunofluorescent images of NeuN levels in the hippocampus regions of NSE-hαSyn Tg mice (*n* = 6, biologically independent animals). Scale bar represents 100 μm. **c** Representative examples of reconstructed hippocampal CA1 pyramidal neurons in NSE-hαSyn Tg mice. **d**, **g** Comparative analysis of the sums of the basal and apical dendrite length and density of pyramidal neurons. **e** Representative examples of hippocampal CA1 pyramidal neurons from the secondary branch in NSE-hαSyn Tg mice. **f** Sholl analysis of reconstructed pyramidal neurons. **h** Comparative analysis of the total number of spines and the numbers of filopodia-like, thin, mushroom, and stubby spines. **i**, **j** Representative immunofluorescent images of SYP and PSD-95 levels in the cortex and CA1 regions of NSE-hαSyn Tg mice (*n* = 6, biologically independent animals). Scale bar represents 20 μm. **k–n** Immunoblot results of SYP, PSD-95, SNAP-25, p-CREB, and CREB levels in the cortex and hippocampus of NSE-hαSyn Tg mice (*n* = 6, biologically independent animals). **o** Representative swimming paths of mice in the MWM test. Representative histogram of the latency to target, target zone time, time in each quadrant, and average speed (*n* = 12, biologically independent animals). The data are presented as the mean ± SD and are representative of three independent experiments performed in triplicate. Significance was determined by using one-way ANOVA with Bonferroni correction; ^#^Comparison between control and NSE-hαSyn Tg mice, *Comparison between NSE-hαSyn Tg mice and osmotin-administered mice. ^#/^*p < 0.05, ^##/^**p < 0.01, and ^###/^***p < 0.001

In previous studies, the overexpression of wild-type human α-synuclein changed dendritic spine density and dynamics [7]. To analyze these effects in response to osmotin treatment, we performed Nissl staining and immunofluorescence in the hippocampus (CA1, CA3, and dentate gyrus). NSE-hαSyn mice showed fewer stained neurons than WT mice. However, this reduction was reversed by osmotin administration (Fig. 5a, b). To analyze the effects of osmotin on α-synuclein-induced alterations in spine morphology, we performed Golgi staining to observe the dendritic structure, complexity, and length in hippocampal CA1 pyramidal neurons (Fig. 5c). We observed a significant decrease in total dendritic length in NSE-hαSyn mice compared with WT mice, and osmotin increased the total dendritic length (Fig. 5d). Additionally, we observed that osmotin significantly increased the dendritic complexity in the basal and apical regions of the NSE-hαSyn mouse brain (Fig. 5e). Furthermore, we examined the spine density in the experimental groups, and the results showed that spine density and the total number of spines in NSE-hαSyn mice were significantly decreased compared to those in WT mice, which were increased by osmotin (Fig. 5f). In particular, the number of filopodia-like spines in the NSE-hαSyn group was significantly reduced compared to that in the WT group, while osmotin increased the spine density, the total number of spines, and filopodia-like spines compared to those in the NSE-hαSyn group (Fig. 5g and h). Interestingly, there were no significant changes in the proportion of thin, mushroom, or stubby spine types. Altogether, our results indicated that osmotin rescued dendritic complexity and spine density in NSE-hαSyn transgenic mice.

### Osmotin alleviates cognitive deficits and rescues synaptic dysfunction in NSE-hαSyn mice

As mentioned before, PD causes motor and nonmotor dysfunctions, including cognitive and synaptic dysfunction [26]. To analyze the protective effects of osmotin against synaptic dysfunction, we investigated synaptophysin (SYP) and postsynaptic density protein-95 (PSD-95). Immunofluorescence results showed a marked decline in the expression levels of SYP and PSD-95 in the frontal cortex and hippocampus (CA1 region) of NSE-hαSyn mice compared to WT mice, and these levels were increased by osmotin treatment (Fig. 5i and j). The results were confirmed by western blot analyses, in which the expression level of SYP, PSD-95, and SNAP-25, as well as the phosphorylation of the memory-associated protein CREB, were significantly reduced in the cortex and hippocampus of NSE-hαSyn mice compared to WT mice. However, osmotin significantly upregulated the expression of these markers in NSE-hαSyn mice (Fig. 5k–n). Last, the MWM results suggested that the percentage of time NSE-hαSyn mice spent in the platform quadrant (zone 2) was significantly decreased compared to that of WT mice. In contrast, osmotin treatment increased their target zone time frequency and time spent in the quadrant (Fig. 5o). Additionally, the latency to reach the platform and the time spent in the target quadrant were significantly improved. The frequency of time spent in the target zone was substantially lower in NSE-hαSyn mice than in WT mice (Fig. 5o). Interestingly, there were no significant differences in the overall average swimming speeds between the experimental groups (Fig. 5o). Taken together, our findings indicated that osmotin attenuated cognitive deficits.

## Discussion

The present study is the first to report that osmotin, an adiponectin receptor agonist, has neuroprotective effects on MPTP/NSE-hαSyn mouse models of PD. Our results suggested that osmotin reduced motor deficits and PD-associated symptoms by regulating TH and its associated factors. Osmotin protected dopaminergic neurons from MPTP/α-synuclein-induced neurotoxicity and alleviated neuroinflammation and apoptotic neuronal cell death. Moreover, osmotin rescued dendritic complexity and cognitive deficits, as indicated by the regulated expression of synaptic markers in NSE-hαSyn mice. The therapeutic effects of osmotin on MPTP/NSE-hαSyn mice correlated with the activation of AMPK via AdipoR1 to block MPTP/α-synuclein-induced susceptibility to PD neuropathology.

The main pathology of PD is the accumulation of α-synuclein in the brain, and phosphorylation of α-synuclein at Ser129 promotes the accumulation of oligomeric and aggregated fibrillar α-synuclein as Lewy bodies [16]. Our study showed that the expression and phosphorylation (Ser129) of α-synuclein, a cardinal feature of PD, was substantially downregulated in NSE-hαSyn mice and A53T-transfected cells after osmotin administration. In addition to α-synuclein, one of the receptors that plays a pivotal role in the pathogenesis of PD is Nurr1, which belongs to the family of ligand-activated transcription factors and plays a role in regulating the morphological and physiological functions of dopaminergic neurons [24]. As a transcription factor, Nurr1 has a known function in activating the TH gene promoter [9]. TH is critical for the formation of L-DOPA, which is the rate-limiting step in the synthesis of dopamine and a precursor of epinephrine and noradrenaline. Therefore, the regulation of TH may protect against the motor deficits induced by α-synuclein in a manner similar to that of the dopamine replacement drug levodopa, which increases dopamine levels in PD patients [46]. Our findings suggested that osmotin modulated Nurr1 and its downstream targets VMAT2, DAT, and TH in MPTP/NSE-hαSyn mice. Furthermore, the TH-related results in osmotin-administered MPTP/NSE-hαSyn mice support our proposed hypothesis about the beneficial effects of osmotin on neurodegeneration.

Additionally, the MAPK family of proteins (i.e., p38, c-JNK, and ERK) are involved in the stress-activated response [8, 25, 29]. Moreover, previous studies have demonstrated that ERK phosphorylation activates Nurr1 and its related factors [34]. In this context, we explored the MPTP/MPP^+^/α-synuclein-induced activation of MAPK family proteins and the possibility that the mechanism of osmotin involves the regulation of these MAPKs, which are involved in the pathophysiology of various neurological diseases [10]. Targeting MAPK inhibition has resulted in neuroprotection against AD and PD [8]. A previous study conducted on the role of osmotin in neurodegenerative diseases suggested that osmotin might counteract the effects of MAPKs and inhibit glutamate-associated cytotoxicity and synaptic dysfunction [44]. Moreover, apoptosis has been reported to be involved in dopaminergic neurodegeneration in studies of postmortem human brains and MPTP-induced animals [55]. In our in vitro studies, cytotoxicity, cell viability, and apoptosis assays indicated the anti-apoptotic effects of osmotin. The inhibition of apoptosis may be due to the inhibition of oxidative stress and the AMPK/MAPK pathways [19]. In a previous study, AMPK-activating agents were used to clear α-synuclein, ameliorate the deficits caused by α-synuclein aggregation, and promote neuronal survival [38]. Similarly, in our study, AMPK phosphorylation was markedly increased in NSE-hαSyn mice and A53T-transfected cells after osmotin administration. Thus, osmotin activates AMPK and MAPK, which regulate dopaminergic neurodegeneration. Moreover, autophagy has an essential role in neuronal homeostasis, acting as a self-degradative process that eliminates misfolded and aggregated proteins through lysosomal degradation [38]. Recently, several studies have suggested that upregulating autophagy is beneficial for reducing α-synuclein aggregation and delaying PD progression [12, 51]. The regulation of autophagy by adiponectin is consistent with the findings of previous studies [33]. To determine the effects of osmotin on autophagy activity, we analyzed the phosphorylation of mTOR, p62, LC3B, and Beclin-1 in A53T-transfected cells, and the results suggested enhanced autophagy activity in response to the administration of osmotin. Our results support previous studies showing that the activation of AMPK regulates autophagy, thereby removing the accumulation of misfolded proteins [12, 38]. We observed that osmotin inhibited mTOR activity via AMPK stimulation, which can induce autophagy pathways. Therefore, osmotin enhanced the clearance of α-synuclein and/or pSer129-α-synuclein. Finally, the other main contributors to neurodegeneration are activated astrocytes and microglial cells, which are critical for releasing inflammatory mediators [2]. In our study, the elevated levels of GFAP and Iba-1 in MPTP/NSE-hαSyn mice were significantly reduced by the administration of osmotin, suggesting the anti-neuroinflammatory effects of osmotin against MPTP/α-synuclein-induced activation of astrocytes and microglia. Elevated oxidative stress may activate astrocytes/microglia, as suggested by the ROS assay results and the expression of NOS-2 and COX-2 in PD models. At this point, it is not clear whether osmotin decreases GFAP and Iba-1 levels via the inhibition of ROS or regulation of AdipoR1.

Although, in general, PD is characterized by motor dysfunction, cognitive decline is another symptom. Dementia has been observed in PD patients, and several studies have investigated cognitive decline associated with PD [15, 52, 54, 56]. Similar to our results, other studies have shown cognitive decline in model mice overexpressing human α-synuclein [5, 32]. A study reported that suppressing α-synuclein levels showed partial clearing of preexisting α-synuclein pathology and improvement in memory through the recovery of structural synaptic defects [32]. Furthermore, studies have shown neuron-glial interaction and its positive effects in dopaminergic transmission, release of neurotrophic factors, and antioxidant productions [37]. Therefore, these may explain how osmotin treatment decreased α-synuclein levels and improved synaptic structure, which may explain the improved cognition. Additionally, NSE-hαSyn mice did not exhibit significantly different swim speeds in the MWM test, showing that dopaminergic neuronal cell death did not affect swim speed. A reasonable explanation for our results concerning the lack of change in motor function is that our PD model mice required less balance maintenance underwater compared to PD patients walking on land [36]. Moreover, our findings show that α-synuclein accumulation causes a reduction in spine density and complexity. These results support other scientific works and suggest that α-synucleinopathy decreases spine density in the cortex, potentially contributing to dementia as a pathophysiological phenotype [27]. Our results showed that osmotin could increase dendritic complexity and length, which may rescue synaptic function in NSE-hαSyn mice. PSD-95 is a postsynaptic scaffold protein that temporally associates with spine morphogenesis through presynaptic dendrites, similar to SYP [40]. Recently, the mechanism of cognitive impairment has been shown to be related to decreases in SYP, PSD-95, and SNAP-25 levels, which may alter the presynaptic integrity triggered by the loss of dopaminergic degeneration [49]. This synaptic dysfunction was significantly reversed by the administration of osmotin, as indicated by the upregulated expression of synaptic markers and the results from the behavioral experiments.

## Conclusions

Collectively, our results suggest that osmotin, a potential adiponectin receptor agonist, can enhance the clearance of α-synuclein and protect against MPTP/α-synuclein-induced neuroinflammation and PD-like pathological neurodegeneration via the AMPK/MAPK pathways in the brains of PD model mice. Therefore, these findings provide insights into the pathways that can be targeted as a therapeutic strategy to protect against PD-associated neurotoxicity and support neuronal protection.

### Supplementary Information

**Additional file 1.** Supplementary table and figures.

## Data Availability

Data generated during the current study are available from the corresponding author upon reasonable request.

## Abbreviations

AdipoR: Adiponectin receptor
AMPK: 5′Adenosine monophosphate-activated protein kinase
BCL-2: B-cell lymphoma 2
CREB: CAMP-response element binding
DAT: Dopamine transporter
ERK1/2: Extracellular signal-regulated protein kinase
GFAP: Glial fibrillary acidic protein
Iba-1: Ionized calcium-binding adapter molecule 1
JNK: C-Jun N-terminal kinase
MAPK: Mitogen-activated protein kinase
MPP^+^: 1-Methyl-4-phenylpyridinium
MPTP: 1-Methyl-4-phenyl-1,2,3,6-tetrahydropyridine
mTOR: Mammalian target of rapamycin
Nurr1: Nuclear receptor-related 1 protein
Os: Osmotin
PD: Parkinson’s disease
PSD-95: Postsynaptic density protein 95
SNAP-25: Synaptosomal-associated protein, 25 kDa
SNpc: Substantia nigra pars compacta
Tg: Transgenic
TH: Tyrosine hydroxylase
VMAT2: Vesicular monoamine transporter 2
