# Supplementary material for: Neuroprotective effects of osmotin in Parkinson’s disease-associated pathology via the AdipoR1/MAPK/AMPK/mTOR signaling pathways
Source: J Biomed Sci. 2023 Aug 11;30:66. doi: 10.1186/s12929-023-00961-z (PMC10422754; doi:10.1186/s12929-023-00961-z)
Supplement: Supplementary file 1 — Additional file 1. Supplementary table and figures. [file 12929_2023_961_MOESM1_ESM.docx]

**Additional Materials**

**Neuroprotective effects of osmotin in Parkinson’s disease-associated pathology via the AdipoR1/MAPK/AMPK/mTOR signaling pathways**

Jun Sung Park^1,†^, Kyonghwan Choe^1,2,†^, Hyeon Jin Lee^1^, Tae Ju Park^3^, Myeong Ok Kim^1,4,#^

**Author affiliations:**

1 Division of Life Sciences and Applied Life Science (BK 21 Four), College of Natural Science, Gyeongsang National University, Jinju 52828, Korea; jsp@gnu.ac.kr (J.S.P.); k.choe@gnu.ac.kr (K.C); lhj4912@gnu.ac.kr (H.J.L.); mokim@gnu.ac.kr (M.O.K);

2 Department of Psychiatry and Neuropsychology, School for Mental Health and Neuroscience (MHeNs), Maastricht University, 6229ER Maastricht, the Netherlands; k.choe@maastrichtuniversity.nl (K.C)

3 Haemato-oncology/Systems Medicine Group, Paul O’Gorman Leukaemia Research Centre, Institute of Cancer Sciences, College of Medical, Veterinary & Life Sciences (MVLS), University of Glasgow, Glasgow G12 0ZD, United Kingdom; t.park.1@research.gla.ac.uk (T.J.P.)

4 Alz-Dementia Korea Co., Jinju 52828, Republic of Korea.

^†^ **These authors contributed equally to this work.**

^#^Corresponding author

Prof. Myeong Ok Kim, Ph.D.

Head of Immune Control Brain Center,

Head of Brain-Metabolic Neurodegenerative Disease Center,

Division of Life Science and Applied Life Science, College of Natural Sciences,

Gyeongsang National University, Jinju, 52828, Republic of Korea

Tel.: +82-55-772-1345, Fax: +82-55-772-2656, E-mail: [mokim@gnu.ac.kr](mailto:mokim@gnu.ac.kr)

**Additional file 1 Figures**

**
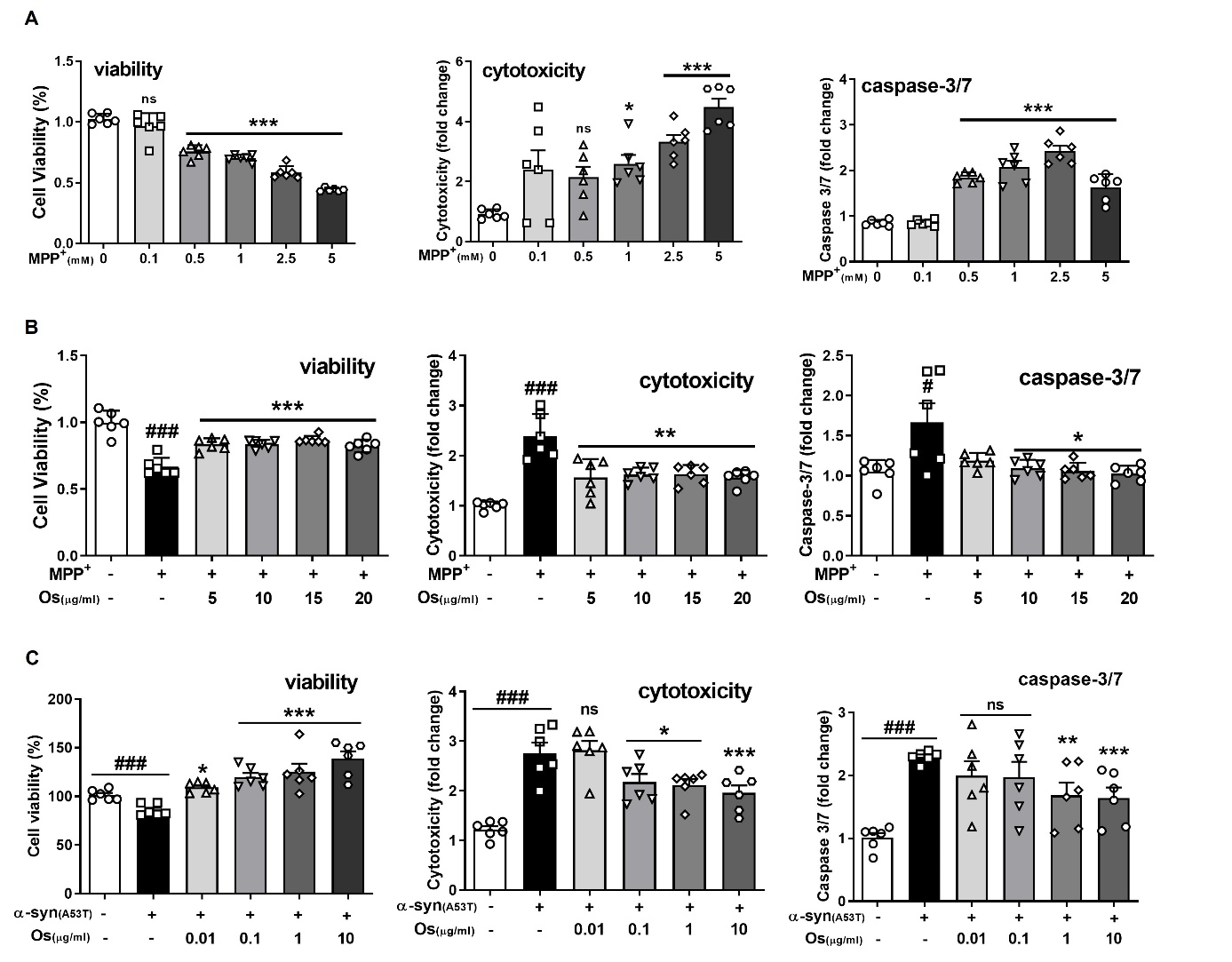
**

**Additional file 1 Figure S1. Osmotin regulates MPTP/α-synuclein-induced cell viability, cytotoxicity, and casepase-3/7 *in vitro*. (A)** Cell viability, cytotoxicity, and caspase-3/7 activity were measured in MPP^+^-induced SH-SY5Y cells. **(B)** Cell viability, cytotoxicity, and caspase-3/7 activity were measured by ApoTox-Glo Triplex assay in MPP^+^-induced SH-SY5Y cells. **(C)** Cell viability, cytotoxicity, and caspase-3/7 activity were examined with an ApoTox-Glo Triplex assay in α-synuclein (A53T)-transfected SH-SY5Y cells. The data are presented as the means ± SD and are representative of three independent experiments performed in triplicate. Significance was determined by using one-way ANOVA with Bonferroni correction; ^#^Comparison between control and MPP^+^/A53T, ^*^Comparison between MPP^+^/A53T and osmotin treated. ^#/*^*p* < 0.05, ^##/**^p < 0.01, and ^###/***^p < 0.001.


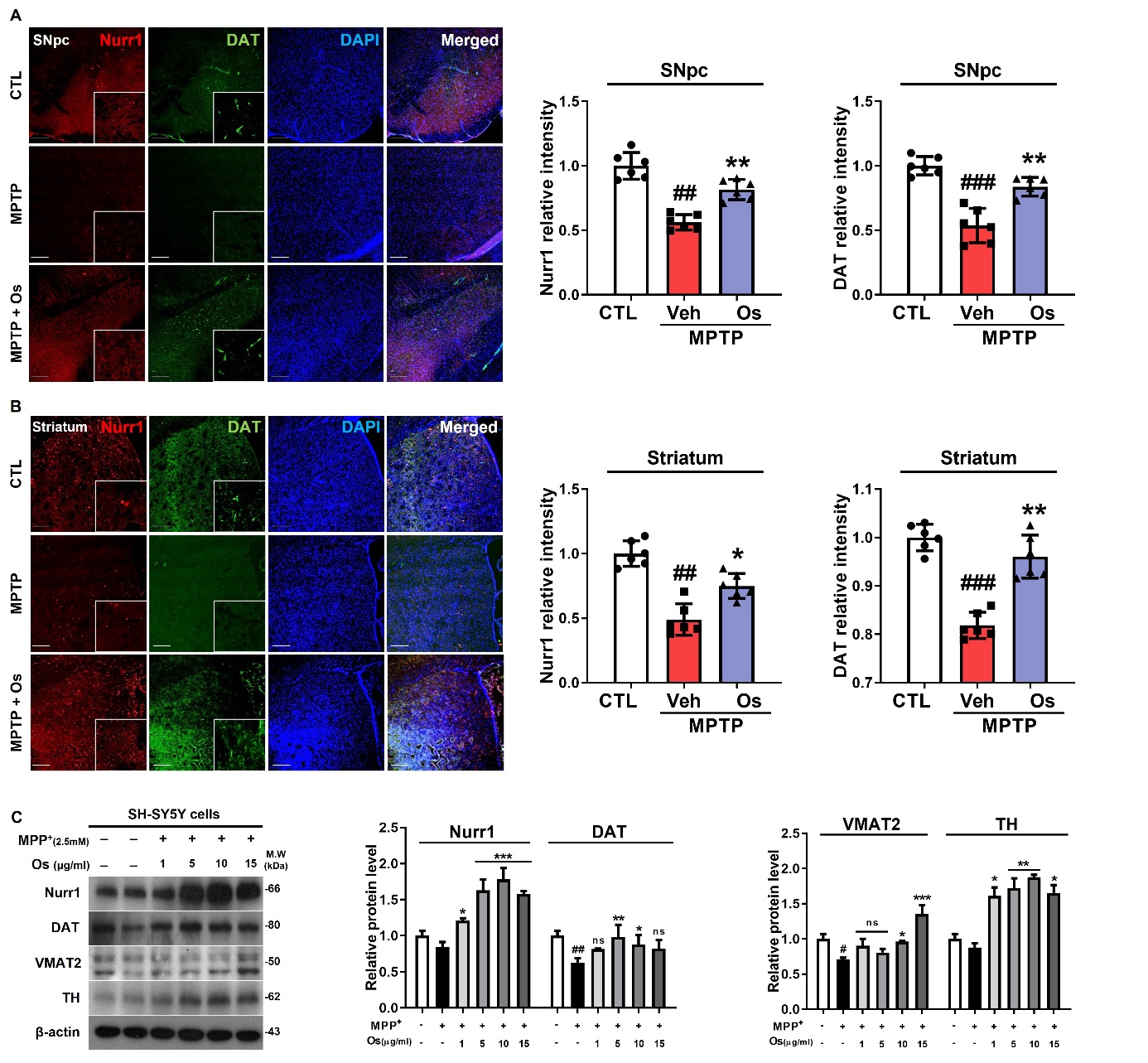


**Additional file 1 Figure S2. Osmotin regulates the expression of Nurr1 and its downstream factors in the MPTP/MPP^+^-induced models. (A)** Representative immunofluorescent images of Nurr1 and DAT in the SNpc from MPTP-induced mice (*n* = 6, biologically independent animals). **(B)** Representative immunofluorescent images of Nurr1 and DAT in the striatum from MPTP-induced mice (*n* = 6, biologically independent animals). **(C)** Immunoblot results of Nurr1, DAT, VMAT2, and TH in MPP^+^-induced SH-SY5Y cells with a graphical representation. Scale bar represents 100μm. The data are presented as the means ± SD and are representative of three independent experiments performed in triplicate. Significance was determined by using one-way ANOVA with Bonferroni correction; ^#^Comparison between control and MPTP/MPP^+^, ^*^Comparison between MPTP/MPP^+^ and osmotin treated. ^#/*^*p* < 0.05, ^##/**^p < 0.01, and ^###/***^p < 0.001.


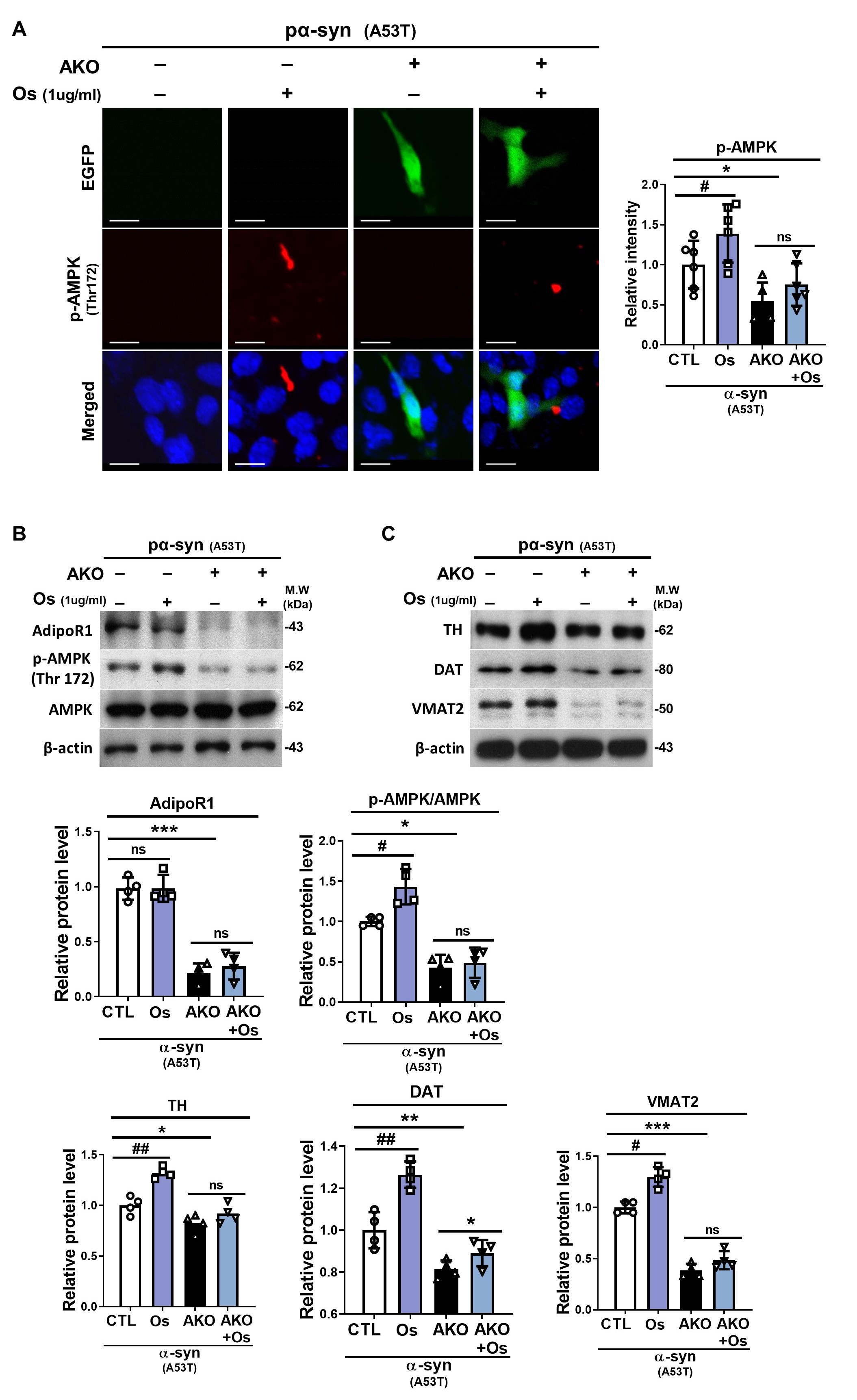


**Additional file 1 Figure S3. Osmotin activates AMPK phosphorylation via AdipoR1 and upregulates TH, DAT and VMAT2. (A)** Representative immunofluorescent images of EGFP and p-AMPK in α-synuclein (A53T)-transfected/adiponectin receptor 1-knockout (AKO) mHippoE-14 cells. **(B, C)** Immunoblot analysis results of AdipoR1, p-AMPK, AMPK, TH, DAT, and VMAT2 in α-synuclein (A53T)-transfected/adiponectin receptor 1-knockout (AKO) mHippoE-14 cells. Scale bar represents 20μm. The data are presented as the means ± SD and are representative of three independent experiments performed in triplicate. Statistical significance was determined by using two-way ANOVA; ^#/*^*p* < 0.05, ^##/**^p < 0.01, and ^###/***^p < 0.001.


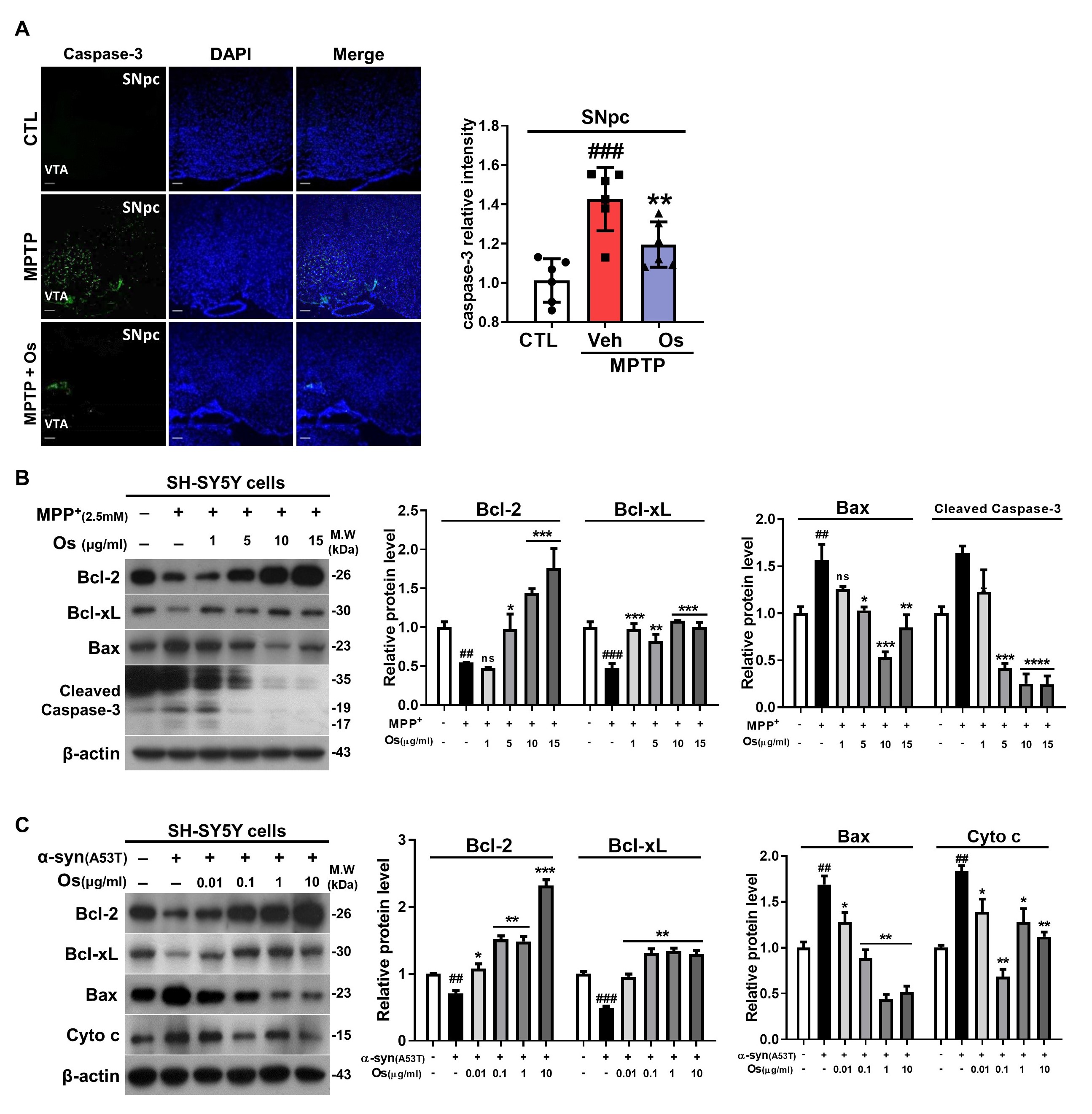


**Additional file 1 Figure S4. Osmotin regulates the expression of apoptotic factors in MPTP/MPP^+^-induced models. (A)** Representative immunofluorescent images of Caspase-3 in the SNpc of MPTP-induced mice (*n* = 6, biologically independent animals). **(B)** Immunoblot results of Bcl-2, Bcl-xL, Bax, and cleaved caspase-3 in MPP^+^-induced SH-SY5Y cells. **(C)** Immunoblot results of Bcl-2, Bcl-xL, Bax, and cytochrome c in α-synuclein (A53T)-transfected SH-SY5Y cells. Scale bar represents 100μm. The data are presented as the means ± SD and are representative of three independent experiments performed in triplicate. Significance was determined by using one-way ANOVA with Bonferroni correction; ^#^Comparison between control and MPTP/MPP^+^/A53T, ^*^Comparison between MPTP/MPP^+^/A53T and osmotin treated. ^#/*^*p* < 0.05, ^##/**^p < 0.01, and ^###/***^p < 0.001.

**
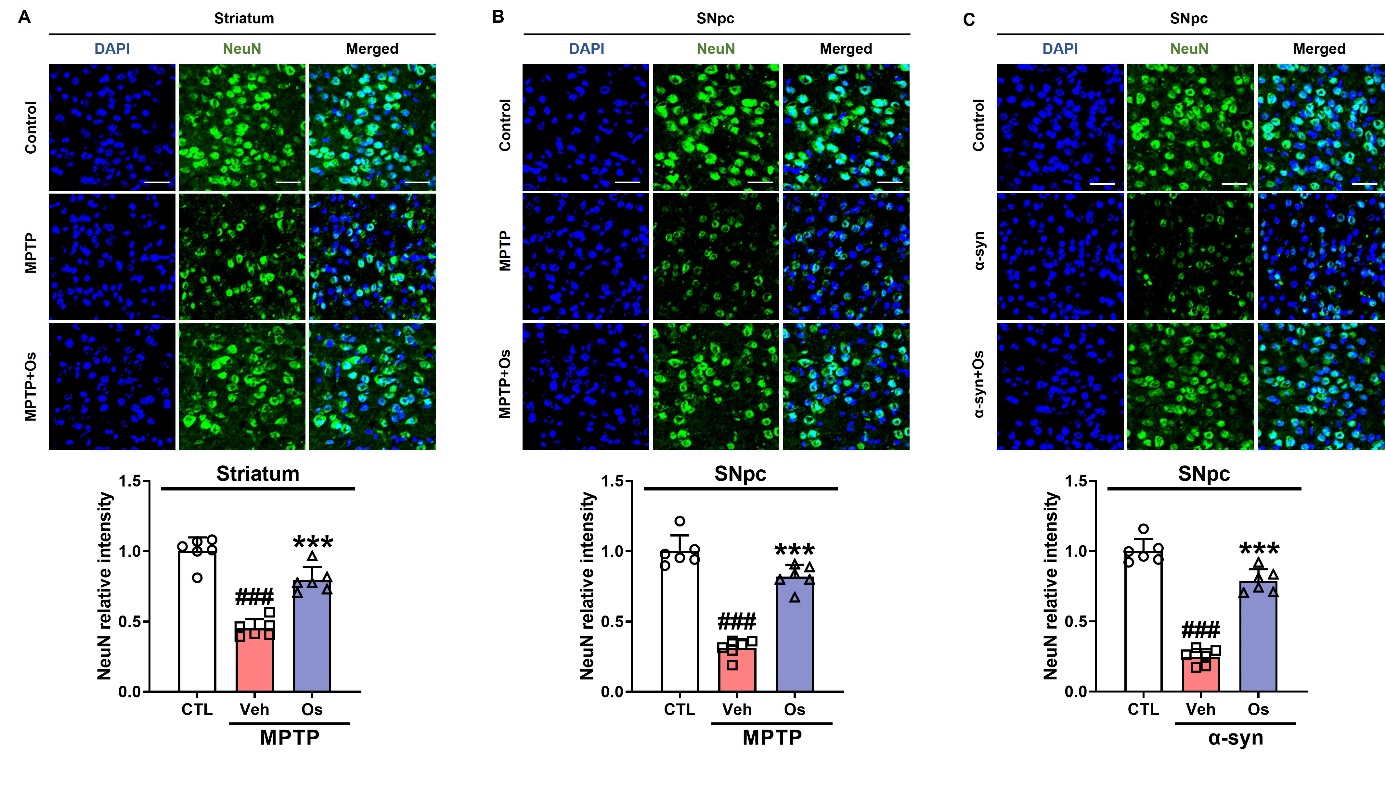
**

**Additional file 1 Figure S5. Osmotin treatment increased the number of Neuron.** Representative immunofluorescence images of NeuN expression in **(A)** striatum and **(B)** SNpc in MPTP mice and **(C)** SNpc in NSE-hαSyn mice. The data are presented as the means ± SD and are representative of three independent experiments performed in triplicate. Scale bar represents 30μm. Significance was determined by using one-way ANOVA with Bonferroni correction; ^#^Comparison between control and MPTP/MPP^+^/A53T, ^*^Comparison between MPTP/MPP^+^/A53T and osmotin treated. ^###/***^p < 0.001.


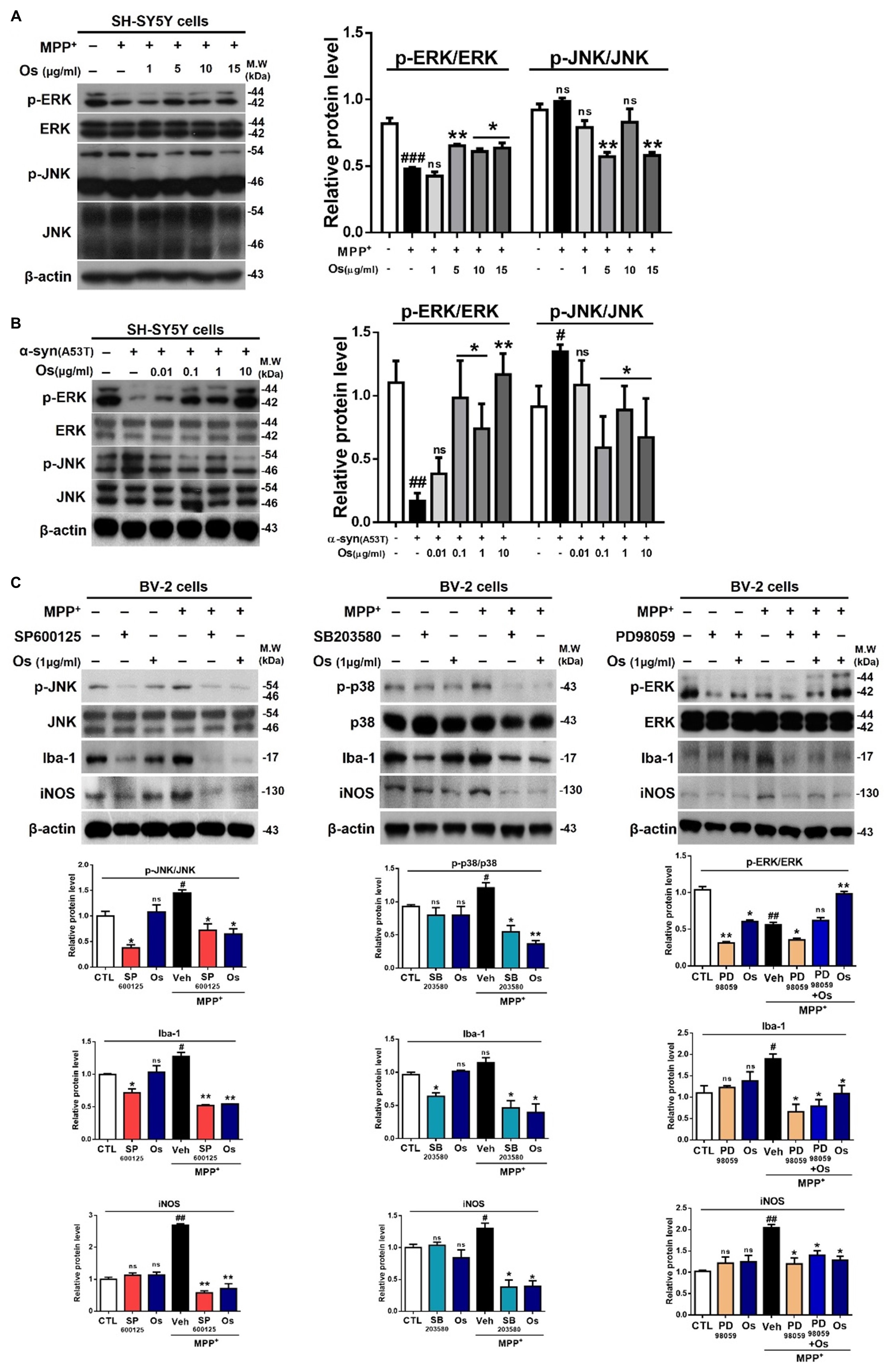


**Additional file 1 Figure S6.** **The effects of osmotin on MAPK signaling pathway in MPP^+^-induced/α-synuclein (A53T)-transfected cells. (A)** Immunoblot results of p-ERK, ERK, p-JNK, and JNK in MPP^+^-induced SH-SY5Y cells with a graphical representation. **(B)** Immunoblot results of p-ERK, ERK, p-JNK, and JNK in α-synuclein (A53T)-transfected SH-SY5Y cells with a graphical representation. **(C)** Immunoblot results under JNK inhibitor (SP600125, 10µM), p38 inhibitor (SB203580, 10µM), and ERK inhibitor (PD98059, 10µM) in BV-2 cells. The data are presented as the means ± SD and are representative of three independent experiments performed in triplicate. Significance was determined by using one-way ANOVA with Bonferroni correction; ^#^Comparison between control and MPP^+^/A53T, ^*^Comparison between MPP^+^/A53T and osmotin treated. ^#/*^*p* < 0.05, ^##/**^p < 0.01, and ^###/***^p < 0.001.


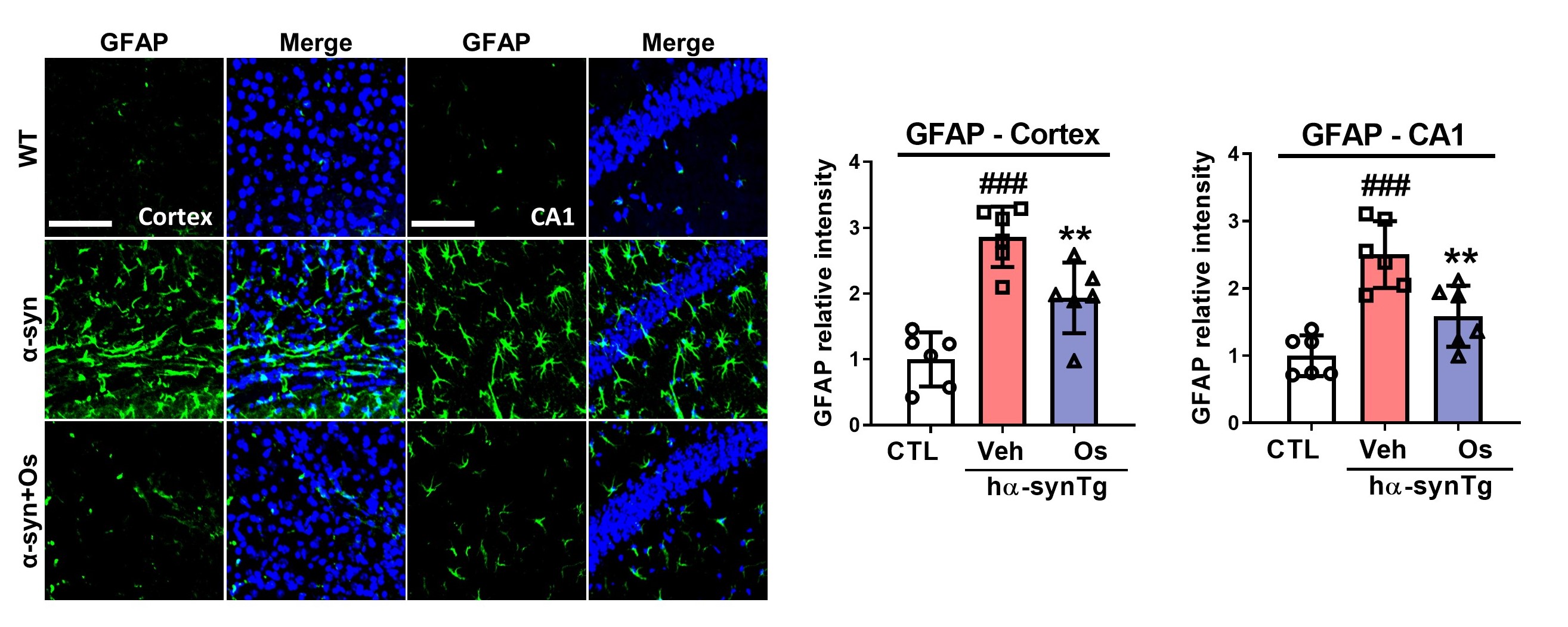


**Additional file 1 Figure S7. Osmotin reduces the expression of GFAP in the frontal cortex and hippocampus (CA1 region) of NSE-hαSyn Tg mice.** Representative immunofluorescent images of GFAP in the cortex and CA1 regions from NSE-hαSyn Tg mice (*n* = 6, biologically independent animals). Scale bar represents 20μm. The data are presented as the means ± SD and are representative of three independent experiments performed in triplicate. Significance was determined by using one-way ANOVA with Bonferroni correction; ^#^Comparison between control and NSE-hαSyn Tg mice, ^*^Comparison between NSE-hαSyn Tg mice and osmotin administrated mice. ^##/**^p < 0.01, and ^###/***^p < 0.001.

**Additional file 1 Table**

**Additional file 1 Table S1**

| Antibodies | Source | Identifier (Cat.) |
| --- | --- | --- |
| Anti-Tyrosine Hydroxylase | **Millipore** | **Cat.#AB152** |
| Anti-NeuN | **Millipore** | **Cat.#MAB377** |
| Anti-LC3B | **abcam** | **Cat.#ab48394** |
| Anti-Beclin 1 | **abcam** | **Cat.#ab62557** |
| Anti-VMAT2 | **abcam** | **Cat.#ab70808** |
| Anti-Alpha-synuclein (phospho S129) | **abcam** | **Cat.#ab51253** |
| Recombinant Anti-Iba1 | **abcam** | **Cat.#ab178846** |
| Anti-Adiponectin Receptor 1 | **abcam** | **Cat.#ab126611** |
| Purified anti-α-Synuclein Phospho (Ser129) | **Biolegend** | **Cat.#MMS-5091** |
| Purified Mouse Anti-Mouse iNOS | **BD biosciences** | **Cat.#610432** |
| Phospho-p44/42 MAPK (Erk1/2) (Thr202/Tyr204) | **Cell Signaling** | **Cat.#9101** |
| p44/42 MAPK (Erk1/2) | **Cell Signaling** | **Cat.#9102** |
| Phospho-p38 MAP Kinase | **Cell Signaling** | **Cat.#9211** |
| p38 MAPK | **Cell Signaling** | **Cat.#9212** |
| mTOR (7C10) | **Cell Signaling** | **Cat.#2983** |
| Phospho-CREB (Ser133) | **Cell Signaling** | **Cat.#9198** |
| Phospho-CREB | **Cell Signaling** | **Cat.#9197** |
| α-Synuclein (D37A6) | **Cell Signaling** | **Cat.#4179** |
| AMPKα | **Cell Signaling** | **Cat.#2532** |
| Phospho-AMPKα (Thr172) | **Cell Signaling** | **Cat.#2535** |
| Cleaved Caspase-3 (Asp175) | **Cell Signaling** | **Cat.#9661** |
| COX-2 | **Santacruz** | **Cat.#sc-7951** |
| GFAP | **Santacruz** | **Cat.#sc-6170** |
| Iba-1 | **Santacruz** | **Cat.#sc-28530** |
| Bax | **Santacruz** | **Cat.#sc-493** |
| Bcl-2 | **Santacruz** | **Cat.#sc-492** |
| Bcl-xL | **Santacruz** | **Cat.#sc-7195** |
| Caspase-3 | **Santacruz** | **Cat.#sc-7272** |
| Cytochrome c | **Santacruz** | **Cat.#sc-13156** |
| PARP-1 | **Santacruz** | **Cat.#sc-8007** |
| DAT (6-8D6) | **Santacruz** | **Cat.#sc-32259** |
| p-mTOR (296.Ser 2481) | **Santacruz** | **Cat.#sc-293132** |
| PSD-95 | **Santacruz** | **Cat.#sc-71933** |
| SYP | **Santacruz** | **Cat.#sc-17750** |
| p-JNK | **Santacruz** | **Cat.#sc-6254** |
| JNK | **Santacruz** | **Cat.#sc-7345** |
| Nurr1 (N-20) | **Santacruz** | **Cat.#sc-991** |
| β-actin | **Santacruz** | **Cat.#sc-47778** |
| Goat anti-Mouse IgG (H+L) Cross-Adsorbed secondary antibody, Alexa Fluor 488 | **ThermoFisher** | **Cat.#A-11001** |

**List of Antibodies.** Representative antibody information, including name, source, and catalog number.
